# Prevalence of Underweight and Its Associated Factors among Reproductive Age Group Women in Ethiopia: Analysis of the 2016 Ethiopian Demographic and Health Survey Data

**DOI:** 10.1155/2020/9718714

**Published:** 2020-07-27

**Authors:** Ayelign Mengesha Kassie, Biruk Beletew Abate, Mesfin Wudu Kassaw, Teshome Gebremeskel Aragie

**Affiliations:** Department of Nursing, College of Health Sciences, Woldia University, P.O. Box 400, Woldia, Ethiopia

## Abstract

**Background:**

Underweight is defined as being below the healthy weight range. Underweight in reproductive age group women not only affects women but also increases the risk of an intergenerational cycle of malnutrition and child mortality. Various factors are linked with underweight among women. However, studies on the prevalence of underweight and its associated factors among women are limited in Ethiopia. Hence, this study aimed to assess the prevalence of underweight and its associated factors among reproductive age group women in Ethiopia.

**Methods:**

For this study, data were drawn from the 2016 Ethiopian demographic and health survey (EDHS). From the total, 15,683 women participants of the 2016 EDHS; a subsample of 2,848 participants aged 15–49 years who had a complete response to all variables of interest were selected and utilized for analysis. Data were analyzed using SPSS version 20 software program. Pearson's chi-squared test was used to assess the frequency distribution of underweight and is presented with different sociodemographic characteristics. Logistic regression models were applied for analysis. A two-sided *p* value of less than 0.05 was used to declare a statistically significant association between the independent variables and underweight among women.

**Results:**

The prevalence of underweight among reproductive age group women in Ethiopia was 17.6%. The majority, 78.3% of underweight women, were rural dwellers. The odds of being underweight was higher among the young aged women, among those residing in rural areas, in those with higher educational status, and in those who have one or more children. On the other hand, the odds of underweight among respondents living in Benishangul, SNNPR, and Addis Ababa were less compared to those living in Dire Dawa. Similarly, the odds of underweight among participants with a higher level of husband or partner educational status and among those who chew Khat were less compared to their counterparts.

**Conclusion:**

Underweight among reproductive age group women in Ethiopia is still a major public health problem, particularly among rural dwellers. Underweight was significantly associated with different sociodemographic variables. Hence, context-based awareness creation programs need to be designed on the prevention methods of underweight in Ethiopia, giving especial emphasis to those residing in rural areas.

## 1. Introduction

Malnutrition can take many forms and represents a large scale and causes complex problems across the world. It affects the majority of the world's population irrespective of location, age, wealth, and gender issues [1]. Many factors like suboptimal diet; food insecurity; poor health status; education; social and gender relations; sociocultural, behavioral, environmental, economic, and political situations; technology; and infrastructure have a great contribution to malnutrition [1, 2].

Underweight is a form of undernutrition and is an indicator of both acute and chronic malnutrition [3]. According to the World Health Organization (WHO) classification, underweight can be defined as a body mass index (BMI) of <18.5 kg/m^2^ for adults and for children and adolescents, the corresponding BMI for age of more than 1 standard deviation below the median of the WHO growth reference for school-aged children and adolescents [4]. Despite the remarkable efforts made on nutrition in both developed and developing countries, approximately 462 million adults were underweight worldwide in 2014 [5].

Unlike men, underweight is more prevalent in women [6]. According to a study in Bangladesh, the women are 2.48 times at higher risk of being underweight than men (36% women vs. 29% men) [7]. Reproductive age group women are particularly vulnerable to undernutrition throughout their life, and lack of adequate weight gain during pregnancy may lead to serious health problems [8, 9]. Furthermore, an undernourished woman is likely to give birth of an underweight child, resulting in the vicious cycle of undernutrition to be repeated over the next generation [10].

The global prevalence of underweight among women has decreased from 14.6% to 9.7% over the past four decades [11]. However, the rate of reduction in underweight is significantly different from country to country. A study in India has revealed that the prevalence of underweight among women has decreased only by 3% from 36% in 1998 to 33% in 2005 [12]. It also varies in different demographic areas within countries and regions [11, 13]. Underweight is higher in women residing in rural areas [14, 15].

It has been reported that information on the prevalence of undernutrition among adults in developing countries is mostly restricted to data on women [16]. In Africa, the prevalence of underweight in countries like Zambia, Zimbabwe, and Niger has decreased significantly. In contrast, in some other countries, like Senegal, Mali, and Madagascar, the rate of underweight is on the rise in both rural and urban areas after a certain period of reduction [17].

According to the 2005 EDHS report, 27% of reproductive age group women in Ethiopia were underweight making their children not only being susceptible to low birth weight, short stature, and low resistance to infections but also increasing the risk of morbidity and mortality rates [17]. This has been reduced to 22% according to the 2016 EDHS [18]. However, this rate of underweight among women in Ethiopia is still higher, and more than double of the global prevalence rate that 9.7% of women are underweight worldwide [11]. It is also in line with the WHO's high prevalence reference rate of 20–29% for underweight [19].

Malnutrition costs the world billions of dollars a year in lost opportunities for economic growth and lost investments in human capital associated with preventable deaths in both children and adults [1, 20]. Low dietary intake is one of the most important risk factors of malnutrition such as primary deficiency due to low levels in the diet and a secondary deficiency due to different diseases interfering with ingestion, absorption, transport, utilization and, or excretion of nutrients [21].

One study in Ethiopia has shown that women's nutritional status is affected by lactation, family planning method utilization, lack of education, illness, and poor dietary habits [22]. However, national studies on the prevalence of underweight and its associated factors among reproductive age group women are limited in Ethiopia. Hence, this study aimed to assess the prevalence of underweight and its associated factors among reproductive age group women in Ethiopia.

## 2. Methods

### 2.1. Study Design and Population

This cross-sectional study was done based on the 2016 EDHS data. The 2016 EDHS was the fourth survey conducted in Ethiopia next to the 2000, 2005, and 2011 surveys. The main aim of the 2016 EDHS was to provide up-to-date information on fertility, childhood mortality, fertility preferences, awareness, approval, and use of family planning methods; maternal and child health; domestic violence; and knowledge and attitude toward HIV/AIDS and other sexually transmitted infections and the prevalence of HIV among the adult population. The survey included representative samples of women (aged 15–49 years) and men (aged 15–59 years) from the nine regions and two administrative cities of the country [18]. However, the current study involved nonpregnant reproductive age group women only because pregnancy nullifies the values of BMI, and data about BMI was not collected among pregnant women and among women who have had a birth in the 2 months before the survey in the 2016 EDHS [18].

### 2.2. Sampling Technique

In the 2016 EDHS, a two-stage stratified sampling technique was employed. In the first stage, the regions in the country were stratified into urban and rural areas. Then, a total of 645 enumeration areas were selected in both urban and rural areas. In the second stage, a fixed number of 28 households per enumeration area were selected with the probability sampling technique. All reproductive age group women who were usual members of the selected households or who spent the night before the survey in the selected households were eligible for the female survey. The details of the sampling process are available elsewhere [18]. For this study, from the total 15,683 women participants of the 2016 EDHS, a subsample of 2,848 reproductive age group women aged 15–49 years who had a complete response to all variables of interest were selected and utilized for analysis after excluding women who were pregnant.

### 2.3. Data Collection

Five standardized and validated questionnaires were used for the 2016 EDHS. The questionnaires were adapted from the DHS Program's standard Demographic and Health Survey questionnaires in a way to reflect the population and health issues relevant to Ethiopia. In addition to the use of validated tools in the data collection process, the 2016 EDHS has used well-trained field personnel and followed standardized protocols to ensure data quality. Data were collected from January 18 to June 27, 2016, with a response rate of 95% for the women's survey [18]. For the purpose of the current study, the women's data from the 2016 EDHS was utilized.

### 2.4. Variables

Several independent variables like respondent's age, education, religion, region, wealth index, and access to media were considered depending on their availability in the 2016 EDHS data. Age was categorized into 3 categories after taking the age group of 15–24 in one group as youth based on the United Nations definition of youth age group [23]. Media access was also classified as yes if the participant had access to at least one of the three public media sources. These are access to magazines/newspapers, listening to the radio and watching television, and no if the participant has no access to all of them. Regarding marital status, according to the 2016 EDHS's definition, women who reported being married or living together with a partner as though married at the time of the survey are considered as ever married [18]. The operational definition of some other variables is available elsewhere [18].

The dependent variable of interest was underweight among nonpregnant ever-married women aged 15–49 years. The outcome variable of interest was categorized based on the WHO Classification of body mass index for adults as follows: underweight if the BMI is <18.5 kg/m^2^ and not underweight if it is ≥18.5 kg/m^2^ [24]. For adolescents aged 15–19 years, the corresponding BMI for age of more than 1 standard deviation below the median of the WHO growth reference for school-aged children and adolescents was used as a cut-off point for underweight [4].

### 2.5. Statistical Analysis

Data analysis started with a summary of the sociodemographic characteristics of women using frequency distribution analysis. Bivariate analysis using Pearson's chi-squared test was used to assess the frequency distribution of the main outcome variable and is presented in relation to different sociodemographic characteristics. Binary logistic regression analysis was done, and variables with a *p* value of less than 0.25 were fitted into the multivariable logistic regression analysis model [25–27]. Then, a multivariable logistic regression analysis was done to examine the association between underweight and the independent variables. A two-sided *p* value of less than 0.05 was used to declare statistically significant odds of association between the independent variables and underweight among women in the multivariable regression model. Data were analyzed using the SPSS version 20 software program.

## 3. Result

### 3.1. Baseline Characteristics of Participants

In this study, 2,848 participants of the 2016 EDHS were included. Around 25% of the participants were within the youth age group classification. More than half, 53.2% of the participants were Orthodox Christian followers, followed by 23.7% Muslims. Regarding residence and level of education, 63% of the participants were from rural areas, 43.2% had no education, and the remaining 56.8% had completed up to higher levels of education. On the other hand, 31.6% of participants' husbands or partners are illiterate and the remaining 68.4% have completed from primary up to higher levels of education. Participants were selected from the nine regions and the 2 administrative cities of the country. Furthermore, more than one-third, 43.6%, of the participants were unemployed and the remaining 56.4% were employed. Concerning wealth index, more than half, 59.6%, of the participants were within the rich wealth quintile (Table 1).

**Figure 1:**
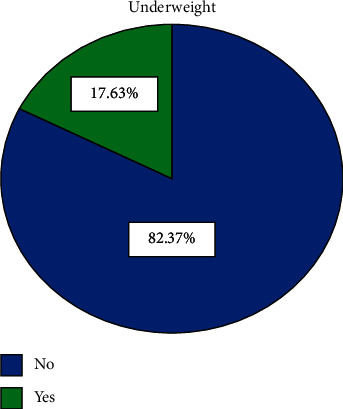
Prevalence of underweight among reproductive age group women in Ethiopia.

The prevalence of underweight among reproductive age group women in Ethiopia was 17.6% (Figure 1). From the total participants who are underweight, the majority, 78.3%, were rural dwellers and the remaining 21.7% were urban dwellers (Table 2).

### 3.2. Factors Associated with Underweight among Women

In this study, bivariate logistic regression analysis was performed, and variables that have a *p* value of less than 0.25 were fitted into the multivariable logistic regression analysis model [25–27]. In the multivariable logistic regression analysis, respondent's age, region, residence, respondent's educational status, husband or partner's educational status, number of children, and Khat chewing were significantly associated with the odds of underweight among women. The odds of underweight among respondents aged 15–24 years was 2.00 (AOR = 2.00, 95% CI (1.38, 2.91) times higher than those aged ≥35 years old. The odds of underweight among respondents living in Benishangul (AOR = 0.45, 95% CI (0.24, 0.85)), SNNPR (AOR = 0.41, 95% CI (0.22, 0.73)), and Addis Ababa (AOR = 0.50, 95% CI (0.27, 0.94)) were less compared to those living in Dire Dawa. Similarly, the odds of being underweight among those who live in rural areas was more than 2.25-fold higher (AOR = 2.25, 95% CI (1.57, 3.23)) than those who live in urban areas.

Educational status was another important variable that was significantly associated with underweight. The odds of underweight among respondents who have secondary (AOR = 1.55, 95% CI (1.02, 2.35)) and higher education (AOR = 2.26, 95% CI (1.24, 4.10)) was higher than those who have no education. Similarly, the husband or partner's educational level was also significantly associated with underweight. The odds of being underweight among respondents with a husband or partner's educational status of a primary level was 1.69 (AOR = 1.69, 95% CI (1.09, 2.63)) times higher than those with a husband or partner having a higher level of education. Besides, the number of children and Khat chewing were also significantly associated with the odds of underweight. The odds of respondents who have 1–3 children (AOR = 1.57, 95% CI (1.04, 2.40)) and those who have 4 or more children (AOR = 1.91, 95% CI (1.14, 3.20)) was higher to be underweight than those who have no children. However, the odds of underweight among respondents who chew Khat (AOR = 1.51, 95% CI (1.02, 2.23)) was lower than those who did not (Table 3).

## 4. Discussion

According to this study, the prevalence of underweight among reproductive age group women in Ethiopia is 17.6%. This finding is in line with studies in India and Nepal which found that 20.1%, and 13.3% of the women were underweight, respectively [28, 29]. From the total participants who are underweight, the majority, 78.3%, were rural dwellers and the remaining 21.7% were urban dwellers. This is also consistent with other study reports in low- and middle-income countries [30]. This might be due to the fact that residing in rural areas is one of the determinant factors which was significantly associated with the prevalence of underweight in this study and other studies [28, 31].

In this study, respondent's age, region, residence, respondent's educational status, husband or partner's educational status, number of children, and Khat chewing were significantly associated with the odds of underweight among reproductive age group women. The odds of underweight among respondents aged 15–24 years was 2.00 (AOR = 2.00, 95% CI (1.38, 2.91)) times higher than those aged ≥35 years old. This finding is in agreement with other studies [28, 30, 31]. This could be due to the fact that the age group consisting of adolescents is a period of rapid physical, psychosocial, and cognitive development with an increased need of nutrients [32]. For example, in South Asia, over 50% of adolescent girls are affected by undernutrition and anemia [33]. It is also reported that undernutrition is high among adolescents living in sub-Saharan Africa including Ethiopia [34, 35]. This might be due to poverty and lack of enough food for consumption because dietary habit is one of the main factors for underweight in adolescents [36, 37].

The odds of underweight among respondents living in Benishangul (AOR = 0.45, 95% CI (0.24, 0.85)), SNNPR (AOR = 0.41, 95% CI (0.22, 0.73)), and Addis Ababa (AOR = 0.50, 95% CI (0.27, 0.94)) was less compared to those living in Dire Dawa. This might be due to socioeconomic and demographic variations because in Ethiopia, for people who are in low socioeconomic status groups and living in rural areas, there are national and international food aids, unlike people who live in urban areas. For example, there is a program called “safety nets” which provides food aid to adult nutrition in rural Ethiopia [38]. Therefore, as Dire Dawa is an urban area, people with low socioeconomic status in Dire Dawa might not get the necessary food aid from national and international aid organizations [39, 40].

Similarly, the odds of being underweight among those who live in rural areas was 2-fold higher (AOR = 2.25, 95% CI (1.57, 3.23)) than those who live in urban areas. This is consistent with other studies [30]. This could be due to differences in educational status, food security, and access to information regarding nutrition education among rural dwellers compared to the rural population [41, 42].

Educational status was another important variable that was significantly associated with underweight. The odds of underweight among respondents who have secondary (AOR = 1.55, 95% CI (1.02, 2.35)) and higher education (AOR = 2.26, 95% CI (1.24, 4.10)) was higher than those who have no education. Similar findings are reported in Kenya and Nigeria [43, 44]. This could be due to the stress education creates on student's nutrition habits resulting in anorexia [45].

However, it is in contrast with other studies which have found that a higher level of education is a risk factor for overweight and obesity in the opposite direction [46, 47]. This inconsistent finding could be due to the fact that occupational stress is one of the major factors which affect body weight in both directions by different mechanisms. On the one hand, stress might result in anorexia and weight loss [45, 48]. On the other hand, it can also increase the secretion of cortisol, a hormone that increases the amount of blood sugar, and might result in increased body weight depending on the individual's response [48].

Similarly, the husband or partner's educational level was also significantly associated with underweight. The odds of being underweight among respondents with a husband or partner educational status of a primary level was around 1.69 times higher (AOR = 1.69, 95% CI (1.09, 2.63)) than those with a husband- or partner-level of higher education. This might be due to the fact that those women with a husband or partner who have higher educational levels may get better support on nutritional issues than those women with a husband or partner of lower education. It might be also due to the sharing of responsibilities in the household [49].

Besides, the number of children and Khat chewing were also significantly associated with the odds of underweight. The odds of respondents who have 1–3 children (AOR = 1.57, 95% CI (1.04, 2.40)) and those who have 4 or more children (AOR = 1.91, 95% CI (1.14, 3.20)) was higher to be underweight than those who have no children. This could be due to an imbalance between the increase in nutrition demand during pregnancy and lactation among mothers who give birth multiple times than those who did not [50].

The odds of underweight among respondents who chew Khat (AOR = 1.51, 95% CI (1.02, 2.23)) was lower by 51% than those who did not (Table 3). This might be due to the effect of Khat that people with a habit of Khat chewing may have a sedentary type of lifestyle, and it may result in weight gain among those who chew it. As an alternative explanation, Khat chewing is a known risk factor for overweight and obesity compared to underweight [51, 52]. A similar finding to the suggested explanation is reported in Ethiopia that Khat chewing increases body weight [53].

### 4.1. Strength and Limitations of the Study

The quality of the data is assured as the EDHS uses well-trained field personnel, a standardized protocol, and validated tools in the data collection process. However, some of the very important determinants of overweight and obesity such as physical activity and dietary habits, air pollution, lack of green space, and walking accessibility were not included in this study because the relevant pieces of information regarding these variables are not available in the 2016 EDHS data [18].

## 5. Conclusion

The prevalence of underweight among reproductive age group nonpregnant women in Ethiopia is significantly high, particularly among rural dwellers. Being within the young age group, residing in rural areas, having higher educational status, and having one or more children were positively associated with the odds of underweight among women. On the other hand, the odds of underweight among respondents living in Benishangul, SNNPR, and Addis Ababa were less compared to those living in Dire Dawa. Similarly, the odds of underweight among participants with a higher level of husband or partner's educational status and among those who chew Khat were less compared to their counterparts. This is worrying because underweight may increase women's vulnerability to different types of problems. Therefore, there is a need to create awareness on prevention and control methods of underweight among women in Ethiopia, giving especial emphasis to those residing in rural areas.

## Figures and Tables

**Table 1:** Sociodemographic characteristics of reproductive age group women in Ethiopia, EDHS 2016 (*N* = 2848).

Characteristics	Response	Frequency	Percentage
Respondents' age	15–24 years old	728	25.6
	25–34 years old	1306	45.9
	≥35 years old	814	28.6

Religion	Orthodox	1516	53.2
	Catholic	21	0.7
	Protestant	623	21.9
	Muslim	674	23.7
	Others	14	0.5

Educational status of respondents	No education	1231	43.2
	Primary	990	34.8
	Secondary	365	12.8
	Higher	262	9.2

Region of respondents	Tigray	330	11.6
	Afar	70	2.5
	Amhara	532	18.7
	Oromia	363	12.7
	Somali	16	0.6
	Benishangul	207	7.3
	SNNPR	480	16.9
	Gambella	192	6.7
	Harari	146	5.1
	Addis Ababa	354	12.4
	Dire Dawa	158	5.5

Area of residence	Urban	1054	37.0
	Rural	1794	63.0

Employment status	Not employed	1243	43.6
	Employed	1605	56.4

Husband/partner's educational status	No education	899	31.6
	Primary	1089	38.2
	Secondary	465	16.3
	Higher	395	13.9

Respondents' wealth index	Poor	698	24.5
	Medium	453	15.9
	Rich	1697	59.6

**Table 2:** Cross-tabulation of underweight with sociodemographic and behavioral factors among women (*N* = 2848).

Respondent characteristics	Underweight		*p* value
	Yes, *n* (%)	No, *n* (%)	
Age			
15–24 years old	173 (34.5)	555 (23.7)	<0.001
25–34 years old	202 (40.2)	1104 (47.1)	
≥35 years old	127 (25.3)	687 (29.3)	
Religion			
Orthodox	260 (51.8)	1256 (53.5%)	<0.001
Catholic	2 (0.4)	19 (0.8)	
Protestant	104 (20.7)	519 (22.1)	
Muslim	134 (26.7)	540 (23.0)	
Others	2 (0.4)	12 (0.5%)	
Number of children			
None	37 (7.4)	202 (8.6)	0.493
1–3	284 (56.6)	1350 (57.5)	
≥4	181 (36.1)	794 (33.8)	
Type of contraceptive use			
Traditional	8 (1.6)	65 (2.8)	0.130
Modern	494 (98.4)	2281 (97.2)	
Duration of current contraceptive use			
≤6 months	294 (58.6)	1372 (58.5)	0.973
>6 months	208 (41.4)	974 (41.5	
Region			
Tigray	94 (18.7)	236 (10.1)	<0.001
Afar	15 (3.0)	55 (2.3)	
Amhara	106 (21.1)	426 (18.2)	
Oromia	75 (14.9)	288 (12.3)	
Somali	5 (1.0)	11 (0.5)	
Benishangul	31 (6.2)	176 (7.5)	
SNNPR	69 (13.7)	411 (17.5)	
Gambella	32 (6.4)	160 (6.8)	
Harari	25 (5.0)	121 (5.2)	
Addis Ababa	23 (4.6)	331 (14.1)	
Dire Dawa	27 (5.4)	131 (5.6)	
Residence			
Urban	109 (21.7)	945 (40.3)	<0.001
Rural	393 (78.3)	1401 (59.7)	
Educational status			
No education	235 (46.8)	996 (42.5)	0.107
Primary	173 (34.5)	817 (34.8)	
Secondary	60 (12.0)	305 (13.0)	
Higher	34 (6.8)	228 (9.7)	
Husband/partner's educational status			
No education	163 (32.5)	736 (31.4)	<0.001
Primary	231 (46.0)	858 (36.6)	
Secondary	60 (12.0)	405 (17.3)	
Higher	48 (9.6%)	347 (14.8)	
Wealth index			
Poor	164 (32.7)	534 (22.8)	<0.001
Medium	103 (20.5)	350 (14.9)	
Rich	235 (46.8)	1462 (62.3)	
Respondent drinks alcohol			
No	290 (57.8)	1302 (55.5)	0.352
Yes	212 (42.2)	1044 (44.5)	
Respondent smokes cigarette			
No	498 (99.2)	2333 (99.4)	0.522
Yes	4 (0.8)	13 (0.6)	
Respondent chews khat			
No	460 (91.6)	2086 (88.9)	0.073
Yes	42 (8.4)	260 (11.1)	
Media access			
No	261 (52.0)	1013 (43.2)	<0.001
Yes	241 (48.0)	1333 (56.8)	
Respondent ever uses the Internet			
No	484 (96.4)	2171 (92.5)	0.002
Yes	18 (3.6)	175 (7.5)	
Source of drinking water			
Unimproved source or not safe drinking water	152 (30.3)	550 (23.4)	0.001
Improved source or safe drinking water	350 (69.7)	1796 (76.6)	

The percentages in Tables 1 and 3 are column percentages and should not be considered as row percentages in interpretation of results.

**Table 3:** Regression analysis of factors associated with underweight among reproductive age group women (*N* = 2848).

Respondent characteristics	Underweight		Bivariate logistic regression	Multivariate logistic regression	*p* value
	Yes, *n* (%)	No, *n* (%)	COR (95% CI)	AOR (95% CI)	
Respondents age					
15–24 years old	173 (34.5)	555 (23.7)	1.69 (1.31, 2.18)	2.00 (1.38, 2.91)	<0.001
25–34 years old	202 (40.2)	1104 (47.1)	0.99 (0.78, 1.26)	1.15 (0.87, 1.53)	0.329
≥35 years old	127 (25.3)	687 (29.3)	1	1	
Religion					
Orthodox	260 (51.8)	1256 (53.5%)	1	1	
Catholic	2 (0.4)	19 (0.8)	0.51 (0.12, 2.21)	0.52 (0.11, 2.36)	0.396
Protestant	104 (20.7)	519 (22.1)	0.97 (0.75, 1.24)	1.04 (0.70, 1.56)	0.818
Muslim	134 (26.7)	540 (23.0)	1.20 (0.95, 1.51)	1.20 (0.83, 1.73)	0.342
Others	2 (0.4)	12 (0.5%)	0.81 (0.18, 3.62)	0.92 (0.20, 4.34)	0.921
Type of contraceptive use					
Traditional	8 (1.6)	65 (2.8)	0.57 (0.27, 1.19)	0.85 (0.39, 1.87)	0.690
Modern	494 (98.4)	2281 (97.2)	1	1	
Respondent's region of residence					
Tigray	94 (18.7)	236 (10.1)	1.93 (1.20, 3.12)	1.25 (0.72, 2.19)	0.429
Afar	15 (3.0)	55 (2.3)	1.32 (0.65, 2.68)	1.23 (0.59, 2.59)	0.585
Amhara	106 (21.1)	426 (18.2)	1.20 (0.76, 1.92)	0.79 (0.46, 1.38)	0.410
Oromia	75 (14.9)	288 (12.3)	1.26 (0.78, 2.05)	0.72 (0.42, 1.25)	0.240
Somali	5 (1.0)	11 (0.5)	2.21 (0.716.86)	1.94 (0.59, 6.41)	0.276
Benishangul	31 (6.2)	176 (7.5)	0.86 (0.49, 1.50)	0.45 (0.24, 0.85)	0.013
SNNPR	69 (13.7)	411 (17.5)	0.82 (0.50, 1.33)	0.41 (0.22, 0.73)	0.003
Gambella	32 (6.4)	160 (6.8)	0.97 (0.55, 1.70)	0.66 (0.35, 1.23)	0.190
Harari	25 (5.0)	121 (5.2)	1.00 (0.55, 1.82)	0.94 (0.50, 1.75)	0.836
Addis Ababa	23 (4.6)	331 (14.1)	0.34 (0.19, 0.61)	0.50 (0.27, 0.94)	0.032
Dire Dawa	27 (5.4)	131 (5.6)	1	1	
Respondent's area of residence					
Urban	109 (21.7)	945 (40.3)	1	1	
Rural	393 (78.3)	1401 (59.7)	2.43 (1.94, 3.05)	2.25 (1.57, 3.23)	<0.001
Respondent's educational status					
No education	235 (46.8)	996 (42.5)	1.58 (1.07, 2.33)	1	
Primary	173 (34.5)	817 (34.8)	1.42 (0.96, 2.11)	1.03 (0.79, 1.34)	0.821
Secondary	60 (12.0)	305 (13.0)	1.32 (0.84, 2.08)	1.55 (1.02, 2.35)	0.039
Higher	34 (6.8)	228 (9.7)	1	2.26 (1.24, 4.10)	0.007
Husband/partner's educational status					
No education	163 (32.5)	736 (31.4)	1.60 (1.13, 2.26)	1.18 (0.73, 1.92)	0.492
Primary	231 (46.0)	858 (36.6)	1.95 (1.39, 2.72)	1.69 (1.09, 2.63)	0.020
Secondary	60 (12.0)	405 (17.3)	1.07 (0.72, 1.61)	1.11 (0.70, 1.77)	0.663
Higher	48 (9.6%)	347 (14.8)	1	1	
Respondent's occupation					
Not employed	235 (46.8)	1008 (43.0)	1.17 (0.96, 1.42)	1.09 (0.88, 1.34)	0.431
Employed	267 (53.2)	1338 (57.0)	1	1	
Wealth index					
Poor	164 (32.7)	534 (22.8)	1.91 (1.53, 2.39)	1.28 (0.97, 1.70)	0.086
Medium	103 (20.5)	350 (14.9)	1.83 (1.41, 2.37)	1.27 (0.93, 1.72)	0.129
Rich	235 (46.8)	1462 (62.3)	1	1	
Number of children					
None	37 (7.4)	202 (8.6)	1	1	
1–3	284 (56.6)	1350 (57.5)	0.92 (0.75, 1.13)	1.57 (1.04, 2.40)	0.033
≥4	181 (36.1)	794 (33.8)	1	1.91 (1.14, 3.20)	0.013
Respondent drinks alcohol					
No	290 (57.8)	1302 (55.5)	1.10 (0.90, 1.33)	1.12 (0.82, 1.53)	0.484
Yes	212 (42.2)	1044 (44.5)	1	1	
Respondent smokes cigarette					
No	498 (99.2)	2333 (99.4)	0.69 (0.23, 2.14)	0.63 (0.19, 2.06)	0.445
Yes	4 (0.8)	13 (0.6)	1	1	
Respondent chews Khat					
No	460 (91.6)	2086 (88.9)	1.37 (0.97, 1.92)	1.51 (1.02, 2.23)	0.040
Yes	42 (8.4)	260 (11.1)	1	1	
Media access					
No	261 (52.0)	1013 (43.2)	1.43 (1.18, 1.73)	1.03 (0.81, 1.32)	0.816
Yes	241 (48.0)	1333 (56.8)	1	1	
Respondent ever uses the Internet					
No	484 (96.4)	2171 (92.5)	2.17 (1.32, 3.56)	1.37 (0.75, 2.49)	0.310
Yes	18 (3.6)	175 (7.5)	1	1	

COR: crude odds ratio. AOR: adjusted odds ratio.

## Data Availability

Data supporting the findings of this article are included within the article.

## Abbreviations

BMI: Body mass index
CI: Confidence interval
EA: Enumeration area
DHS: Demographic and health surveys
EDHS: Ethiopia Demographic and Health Survey
ICF: Inner city fund
OR: Odds ratio
SNNPR: Southern Nations, Nationalities, and Peoples' Region
WHO: World Health Organization.
